# Supplementary material for: Correlating dynamic strain and photoluminescence of solid-state defects with stroboscopic x-ray diffraction microscopy
Source: Nat Commun. 2019 Jul 29;10:3386. doi: 10.1038/s41467-019-11365-9 (PMC6662806; doi:10.1038/s41467-019-11365-9)
Supplement: Supplementary file 1 — Supplementary Information [file 41467_2019_11365_MOESM1_ESM.pdf]

Supplementary Information for:

Correlating dynamic strain and photoluminescence of solid-state defects with stroboscopic x-ray diffraction microscopy

S. J. Whiteley,[1,2] F. J. Heremans,[1,3] G. Wolfowicz,[1,3,4] D. D. Awschalom,[1,3] and M. V. Holt[5,*]

[1]*Pritzker School of Molecular Engineering, University of Chicago, Chicago, Illinois 60637, USA*

[2]*Department of Physics, University of Chicago, Chicago, Illinois 60637, USA*

[3]*Center for Molecular Engineering and Materials Science Division, Argonne National Laboratory, Lemont, Illinois 60439, USA*

[4]*WPI-Advanced Institute for Materials Research (WPI-AIMR), Tohoku University, Japan*

[5]*Center for Nanoscale Materials, Argonne National Laboratory, Lemont, Illinois 60439, USA*

* mvholt@anl.gov

I. EXPERIMENTAL SETUP

A. Electronic schematic

The stroboscopic Scanning X-ray Diffraction Microscopy experiment uses a radio frequency (RF) signal generator matched to a multiple of the Advanced Photon Source (APS) synchrotron storage ring frequency (f_{ring}) to drive the interdigitating transducer device. The ring frequency in Fig. 2 is calibrated to be roughly $f_{ring} = 351,934,790$ Hz with a long term drift of a few Hz per hour and a short term drift in a random walk of < 0.1 Hz/s. Over a few months, we found that f_{ring} can changed less than 20 Hz. As the current synchronization signal from the APS control room in this experiment is too jittery to use directly as a trigger input, the signal generator is manually set to f_{ring} with a reference and connection to the APS control room via an ethernet connection. This allows for independent control over the center frequency (f_0) while continuously obtaining appropriate offsets to remain stroboscopic with respect to the storage ring timing. The frequency offset from the APS control room is accurate to within 0.1 Hz and mitigates most of the long term drift of the ring frequency. The short term drift, from APS doing feedback and adjusting the ring frequency, as well as the inability to trigger the RF signal directly causes variations in the phase delays, which currently limits this imaging technique to only to non-propagating acoustics. Improved fiber connections from the APS control room to the beamline are planned to improve the timing accuracy in order to allow for full phase control imaging of the acoustic waves.

From the signal generator (SRS SG396), the RF signal goes to an Amplifier (Minicircuits ZHL-20w-13s+), a directional coupler (Fairview Microwave MC51008-20), and finally a RF circulator (Fairview Microwave SFC3340S) before entering the vacuum chamber to the sample. The -20 dB coupling port from the directional coupler is connected to a zero-biased Schottky diode (Fairview Microwave SMD0112) to measure the RF power at the sample, and the reflections from the sample are sent via the circulator to an oscilloscope to verify the RF timing with the APS ring, and X-ray experiment. Typical RF power at sample was 27-30 dBm. An RF electronic schematic of the experiments device is shown in Supplementary Figure 1.

B. Layout of Hard X-ray Nanoprobe

The CNM/APS Hard X-ray Nanoprobe (HXN) facility at beamline 26-ID of the Advanced Photon Source (APS), Argonne National Laboratory (ANL) delivers a hard X-ray beam tunable

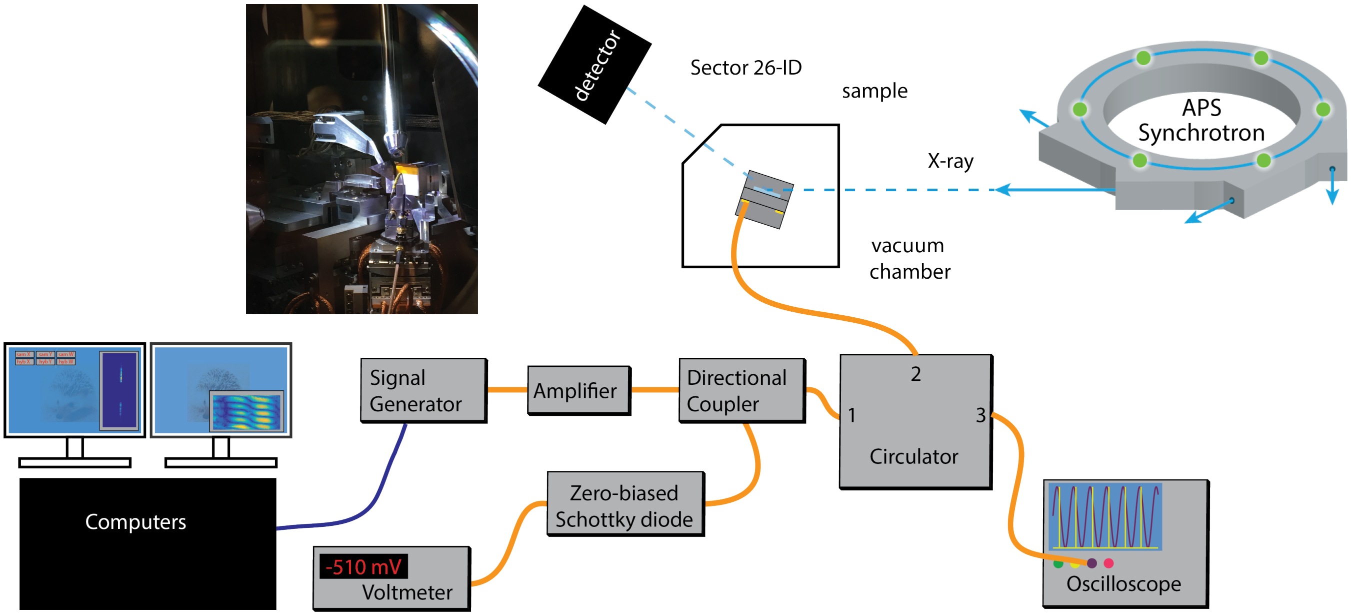

Supplementary Figure 1. **Electronic schematic of the stroboscopic experiment.** Matching the frequency of the X-ray bunches from the Advanced Photon Source with the signal generator requires setting the frequency of the signal generator with a real-time frequency offset using a reference from the APS control room. The RF signal is then amplified, coupled to a Schottky diode, and sent through a circulator before entering the sample chamber. The Schottky diode is used to verify the approximate RF power at the device and a circulator minimizes back reflections along with sends the outgoing signal to an oscilloscope.

over the 6-12 keV spectral range and focused to 25 nm spot size in-plane onto the sample. The HXN uses interferometric control to maintain relative positional drift of the focusing optics and sample less than 10 nm/h. The working distance between the X-ray focusing optics and the sample is typically a few millimeters. This enables a variety of *in situ* and *operando* experiments with variable temperature, applied electric and magnetic fields, and liquid and gaseous environments. Nanoscale structural information, such as crystallographic phase, strain, and texture, are measured at the HXN at a $\sim$25 nm real-space spatial resolution by recording how a crystalline sample diffracts the incident nanofocused X-ray beam while on the Bragg condition as the focus is scanned over the sample. Bragg ptychography, a scanning coherent diffraction imaging technique that exploits the coherence of the nanofocused x-ray beam combined with iterative phase retrieval methods, provides nanoscale structure and lattice strain information within crystalline samples at a demonstrated resolution extending to 5 nm, well beyond the resolution of current hard X-ray focusing optics. Both the scanning nanodiffraction methods and future, time-resolved studies with the HXN will provide new abilities tools for probing acoustic interactions with crystal ordering, defects, and phase transitions in nanomaterials.

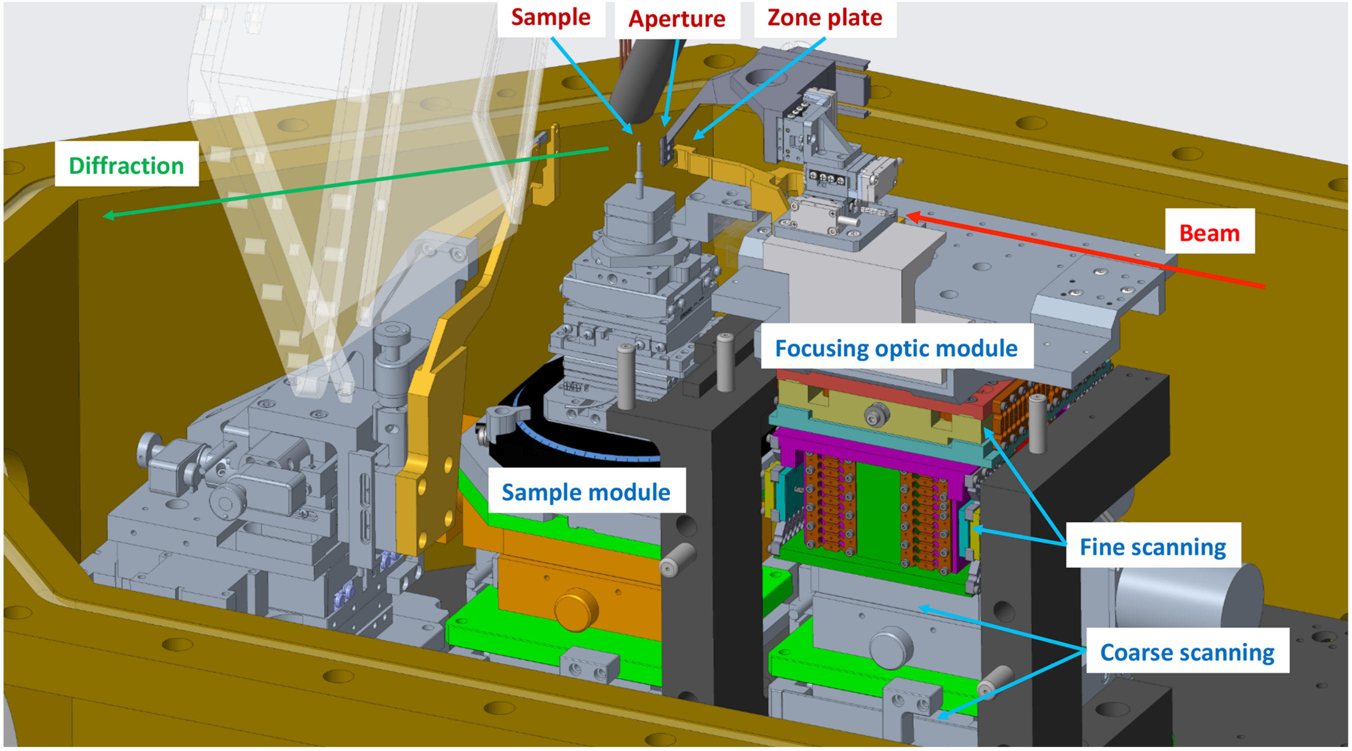

Supplementary Figure 2. **Nanoprobe chamber and instrument schematic.** X-rays from the Advanced Photon Source are downselected to a monochromatic coherent fraction prior to being focused onto the sample by a hard X-ray Fresnel zone plate focusing optic. The sample scanning directions are vertically upwards and downstream (along the X-ray beam, in the plane of the sample surface) - rotating about the X-ray downstream vector at the zero diffraction angle.

II. MICROFABRICATION

The 4” diameter 4H silicon carbide (SiC) wafer had $\sim$ 500 nm of aluminum nitride (AlN) sputtered on the surface by OEM Group Inc. The AlN film had a X-ray diffraction rocking curve of 1.52° FWHM on the [0002] peak and the film stress was -42 MPa. All device layers were fabricated by optical lithography and dry etching with inductively coupled plasma (ICP) in a PlasmaTherm Apex SLR.

The IDT device layer was formed by electron-beam evaporation with 20 nm of titanium (Ti) and 150 nm of aluminum (Al) using an Angstrom EvoVac. The Ti serves both as an adhesion layer for the Al and as a luminescent layer for X-ray fluorescence mapping. Optical lithography, utilizing a 0.9 μm thick photoresist, for the interdigitated transducer (IDT) pattern was subsequently performed using a GCA AutoStep 200. To direct write the autostepper reticle for this process, we used a Heidelberg MLA150. The IDT pattern was transfered to the Ti/Al metal device layer

by ICP etching with $Cl_2/BCl_3/Ar$ with flow rates of 30/30/10 sccm, respectively, and 400 W ICP power. Next, we define a ”film window” at the acoustic beam waist in the AlN piezoelectric transduction layer by direct-write optical lithography, using the Heidelberg MLA150. This releases inhomogeneous SiC strain caused by the AlN. ICP etching of the AlN film window was accomplished using $Cl_2/BCl_3/Ar$ with flow rates of 30/30/10 sccm, respectively, and 400 W ICP power. Finally, direct write optical lithography for the circular pit employed a ~ 2.9 μm thick photoresist and purposefully over-exposing a 1 μm circle. The circular pit pattern was transferred to the 4H-SiC by ICP etching using SF_6/Ar with flow rates of 40/10 sccm, respectively, and 500 W ICP power. We characterized the etched pit depth by atomic force microscopy (AFM) measurements (Supplementary Figure 3). Our AFM measurements also found that the SiC surface had up to 5 nm of increased roughness after ICP etching, which was transferred from the rougher AlN film when fabricating the film window.

III. DIVACANCY PHOTOLUMINESCENCE RESPONSE

Optically-active point defects in the 4H-SiC substrate provide numerous spin and electronic properties, sensitive to environmental perturbations. While the ground state spins of point defects, including the neutral divacancy (VV^0) and negatively charged silicon vacancy (V_{Si}^-), in SiC can be used to sense small magnetic fields [1], their charge state provides a straightforward means of measuring and imaging internal electric fields [2]. Here, we use the technique Electrometry by Optical Charge Conversion (EOCC) on native VV defect ensembles in the SiC substrate as local sensors of mechanics. In our implementation of EOCC, we use two laser colors on VV ensembles: 405 nm to perform charge state resets and 976 nm for optical charge conversion as well as read out. While illuminating with 976 nm, the rate of optical charge conversion, detected by photoluminescence (PL) from the VV on a photodiode, is dependent on the radio frequency electric field at the VV defects. Since SiC is piezoelectric, bulk strain will generate an internal electric field and be detected in three dimensions via EOCC.

In the absence of RF driving of the Gaussian SAW, PL from the VV ensembles is spatially homogeneous over the SiC in the region where an AlN was removed to create a film window (Supplementary Figure 4a). Over the AlN, we observe that the PL is reduced by $\approx 70\%$, which is likely due to lower optical transmission through the sputtered polycrystalline AlN layer. An overview map of EOCC (Supplementary Figure 4a) yields ’hot spots’ near the electrodes and film

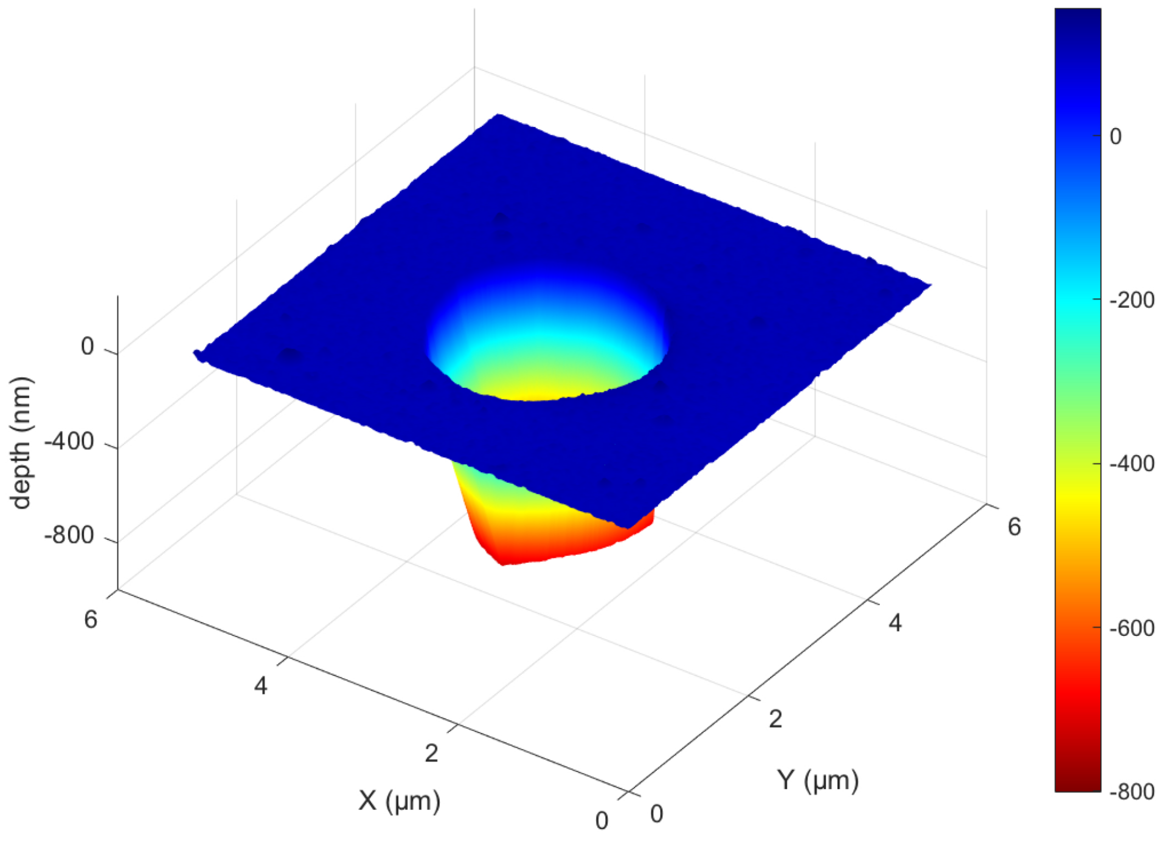

Supplementary Figure 3. **Atomic force microscopy (AFM) measurements of the etch pit.** The pit depth is ∼ 950 nm deep. This is the same sample and pit that is shown in Figs. 3,4. Since the AFM tip's triangular shape distorts the in-plane dimensions, the SEM images in Fig. 3a should be consulted for the pit shape and diameter. The color scale is in nanometers.

window edge, along with a gradient across the device center originating from the RF side - we attribute all of these EOCC features to stray electric fields from the IDT and RF drive. EOCC from the SAW far away from the pit is too weak to be measured on top of the background from stray electric fields.

Next, we zoom in near the pit and take finer in-plane images of PL and EOCC contrast (Supplementary Figure 4b). Even though the pit walls cause laser scattering and induce an optical shadow, the EOCC map reveals two maxima spatially resolved near the longitudinal ($\pm\boldsymbol{y}$) pit edges. Therefore, the SAW standing wave generates dynamic strains near the structural defect edges in the direction of acoustic propagation. Additionally, the EOCC contrast near the pit qualitatively agrees with s-SXDM results (Fig. 4), which show the structural defect's dynamic strain is enhanced over the regular Gaussian SAW mode. The background EOCC contrast gradient from

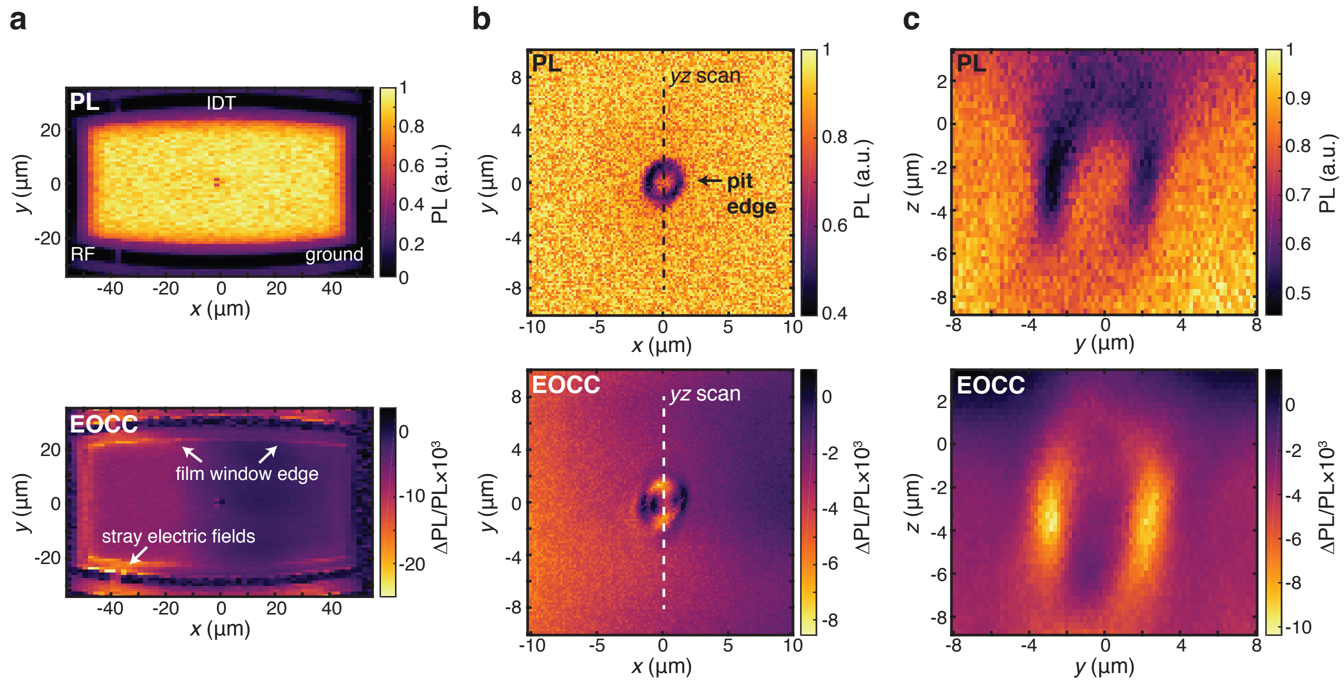

Supplementary Figure 4. **Divacancy photoluminescence (PL) mapping and electrometry near the fabricated structural defect.** (**a**) Overview (*xy* map) of the Gaussian SAW beam waist region of the device. (**b**) Scan (*xy* map) near the etched pit. A reduction in PL near the pit is slightly visible in a 2 μm radius about $(x, y) = (0, 0)$. EOCC mapping shows two lobes of contrast (reduced PL during SAW driving) near the longitudinal edges of the pit. (**c**) Depth cross-sectional scan (*xz* map) through the pit at the dashed line in (**b**). In all panels the upper image is PL from divacancy defects in the SiC substrate, and the lower image is continuous-wave EOCC of the divacancies. The *x*, *y*, and *z* directions correspond to longitudinal, transverse, and sample c-axis directions, matched to sample orientations in the s-SXDM experiments.

stray RF electric fields is visible even micrometers away from the pit (Supplementary Figure 4b). From depth cross-sectional scans, we find that the two lobes of EOCC contrast are maximized beneath the SiC surface and correlate to lower pit corners, in agreement with the phenomenological simulation in the main text Fig. 3c.

In order to calibrate the EOCC signal, we measure the EOCC response in the capacitor gaps (center pin to ground) formed by the input coplanar waveguide near the Gaussian IDT (Fig. 1a). The calibration is obtained by combining the EOCC contrast as a function of input microwave power (Supplementary Figure 5) with finite-element (COMSOL) simulations of the electric field. In turn, we estimate that the peak electric field measured near the pit is approximately 300 V/cm at 400 mW of drive power after background corrections. Using knowledge of strain and electric field distributions in the 4H-SiC caused by mechanics (Supplementary Figs. 6b and 7), we can convert the estimated peak electric field of 300 V/cm to an estimated strain amplitude. We can see

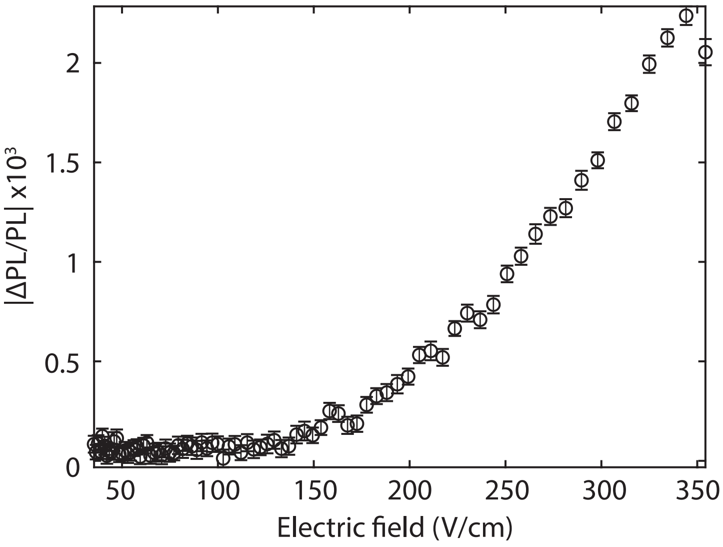

Supplementary Figure 5. **Sensitivity calibration for electrometry by optical charge conversion (EOCC).** Measured magnitude of EOCC optical contrast as a function of linear electric field amplitude (converted from input power) in between the coplanar waveguide gap. Error bars are one standard deviation.

that electric field and strain scale together linearly from the piezoelectric equations (stress-charge form),

$$\begin{aligned} \boldsymbol{\sigma} &= \mathbf{c}^{\mathrm{E}}\mathbf{S} - \mathbf{e}\mathbf{E}\,, \\ \mathbf{D} &= \boldsymbol{\epsilon}\mathbf{E} + \mathbf{e}\mathbf{S}\,, \end{aligned} \tag{1}$$

where the tensors $\boldsymbol{\sigma}$ is stress, $\mathbf{S}$ is strain, $\mathbf{D}$ is the electric displacement field, $\mathbf{E}$ is the electric field, $\boldsymbol{\epsilon}$ is the matrix of dielectric constants, and $\mathbf{c}^{\mathrm{E}}$ is the elasticity (i.e. stiffness tensor). Note that normally we use $\varepsilon_{ij} = \frac{1}{2}(\frac{\partial u_i}{\partial x_j} + \frac{\partial u_j}{\partial x_i})$ in the paper and supplement to represent strain, although this equation shows $\mathbf{S}$ to distinguish the variables more easily. By applying a linear scale factor found from COMSOL simulations in the time-domain, we find the estimated uniaxial strain near the pit from 400 mW of RF drive power is $\varepsilon_{zz} = 8.3 \times 10^{-5} \pm 4.7 \times 10^{-5}$. The uncertainty in this estimation in part comes from which spatial locations in the simulation are used for the linear scale factor, therefore, one the dynamic strain near the pit corner should be treated as 10^{-4} order of magnitude.

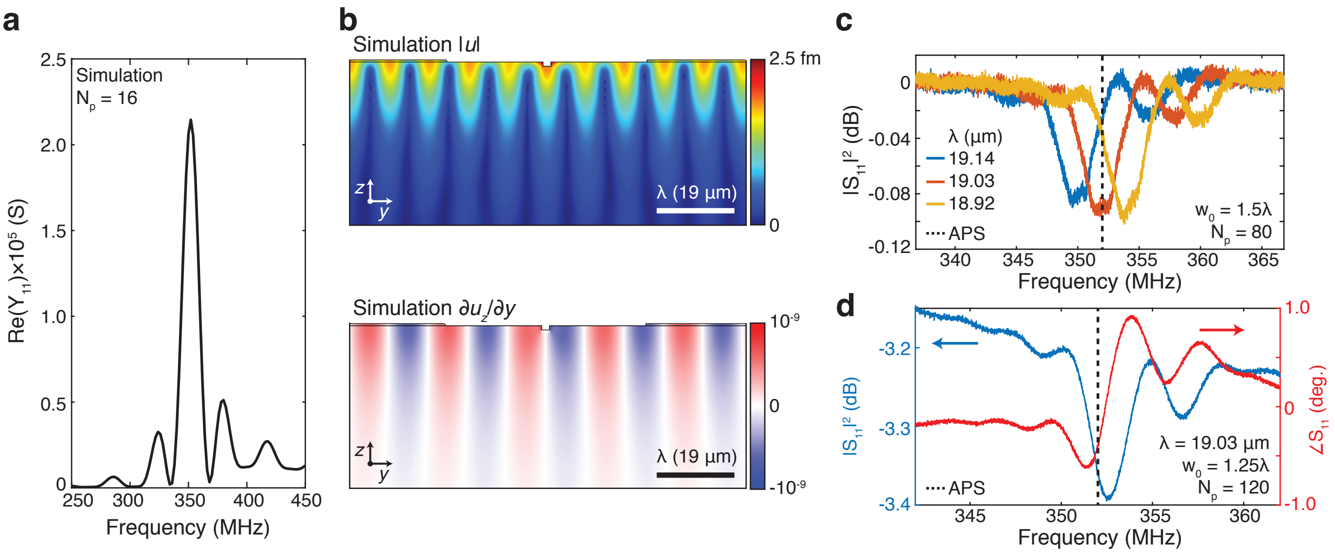

Supplementary Figure 6. **RF characterization of Gaussian IDT.** (**a**) Frequency domain simulated admittance of a planar IDT using COMSOL Multiphysics 2D (yz) cross-section. The simulation accurately predicts the IDT center frequency (352 MHz), and therefore acoustic velocity $v_{SAW} = \lambda f_0$, for a wavelength of 19.03 μm. (**b**) Time domain simulated absolute displacement (upper image) and longitudinal lattice slope (lower image). The applied IDT voltage is 1 mV. The simulations confirm that the etched film window have a negligible effect on the standing wave mode and displacements are enhanced at the structural defect walls and corners. (**c**) RF reflection measurements for Gaussian IDTs of varying the acoustic wavelength, plotted with subtracted magnitude offsets. While keeping the Gaussian geometry factors (e.g. spot size w and electrode finger pairs Np) constant, the IDT center frequency f_0 is easily tunable by changing λ. (**d**) RF measurement of the Gaussian IDT used in s-SXDM experiments, wirebonded to a printed circuit board. The Gaussian IDT geometry appears to induce an asymmetric relative amplitude of the sinc-squared side peaks. Sub-MHz errors in f_0 may come from non-uniform AlN thickness across the wafer. The target frequency of Advanced Photon Source (APS) synchrotron timing structure is shown (black dashed line) for convenience.

IV. SAW CHARACTERIZATION AND MODELING

In order to find the proper SAW wavelength (λ) for s-SXDM experiments, we first by fabricate planar, single transducer planar IDTs (no Gaussian focusing) using single layer lithography. By attempting various acoustic wavelengths, such as 8, 12, 16 μm (not shown), and performing RF characterization with a vector network analyzer (VNA), we can estimate that we require an IDT with $\lambda \sim 19 \pm 0.1$ μm to get a center frequency $f_0 \sim 352$ MHz. This agrees with an planar IDT simulation (Supplementary Figure 6a). Simulated 2D cross-sections (yz) of Rayleigh wave produced at this f_0 and λ are shown in Supplementary Figure 6b, including the structural defect and AlN film window. Between multiple rounds of device fabrication, we find that chips from the middle of the wafer and 1 cm from the wafer's edge (containing the same IDT designs) produce

center frequency variations $\leq 0.3\%$. This suggests that the effective Rayleigh waves velocities between devices are reproducible and the AlN film thickness is sufficiently uniform across the wafer.

The high degree of device repeatability and AlN thickness uniformity allows us to easily fabricate many IDTs and vary their geometry until the frequency performance is optimal for s-SXDM. We fabricate Gaussian IDTs ($w_0 = 1.5\lambda$ and 80 electrode finger pairs) and vary the acoustic wavelength λ in the mask design between devices by 11 nm. Even though optical lithography has limited resolution for a single feature, the increments in electrode periodicity work well enough to experimentally find the λ needed for s-SXDM (Supplementary Figure 6c). Using $\lambda = 19.03$ μm for s-SXDM, we fabricate Gaussian IDTS with extra electrodes for improved SAW admittance and a smaller SAW focus ($w_0 = 1.25\lambda$). Small structural defects, like an etch pit, had little to no impact on RF reflection results. The 1 port reflection measurement with VNA for the device used in experiments is shown in Supplementary Figure 6d. The IDT could not be impedance matched to 50 Ω because AlN and SiC are strong piezoelectric substrates, and we did not want to use even more electrode finger pairs, which could have risked making the IDT bandwidth too narrow and cause f_0 to miss the APS synchrotron bunch frequency.

We show a full three-dimensional simulation of a SAW wave interacting with a pit in Supplementary Figure 7. This simulation uses approximately planar (non-curved) electrodes with an aperture similar to the Gaussian acoustic focal spot, and a symmetry plane is used at $x = 0$ because the device design is in fact symmetric. All scalebar values are from the simulation and can be globally, linearly scaled together in order to compare to experimental values (1 nm maximum displacement expected in experiments). The strain distributions and E^2 field from the SAW in this model qualitatively agreed with the 2D model shown in the main text (Fig. 3). EOCC enhanced by the pit is expected to be detected on the longitudinal ($\pm y$) sides of the pit at the lower corner from (Supplementary Figure 7d,e). Curvature (i.e. variations in lattice slope) enhancements are predicted from the simulation (Supplementary Figure 7c), however, this effect is much more noticeable in the transverse curvature map near the pit (Supplementary Figure 7f) because there is a node in $\frac{\partial u_z}{\partial y}$ at $x = 0$ due to the mirror plane symmetry.

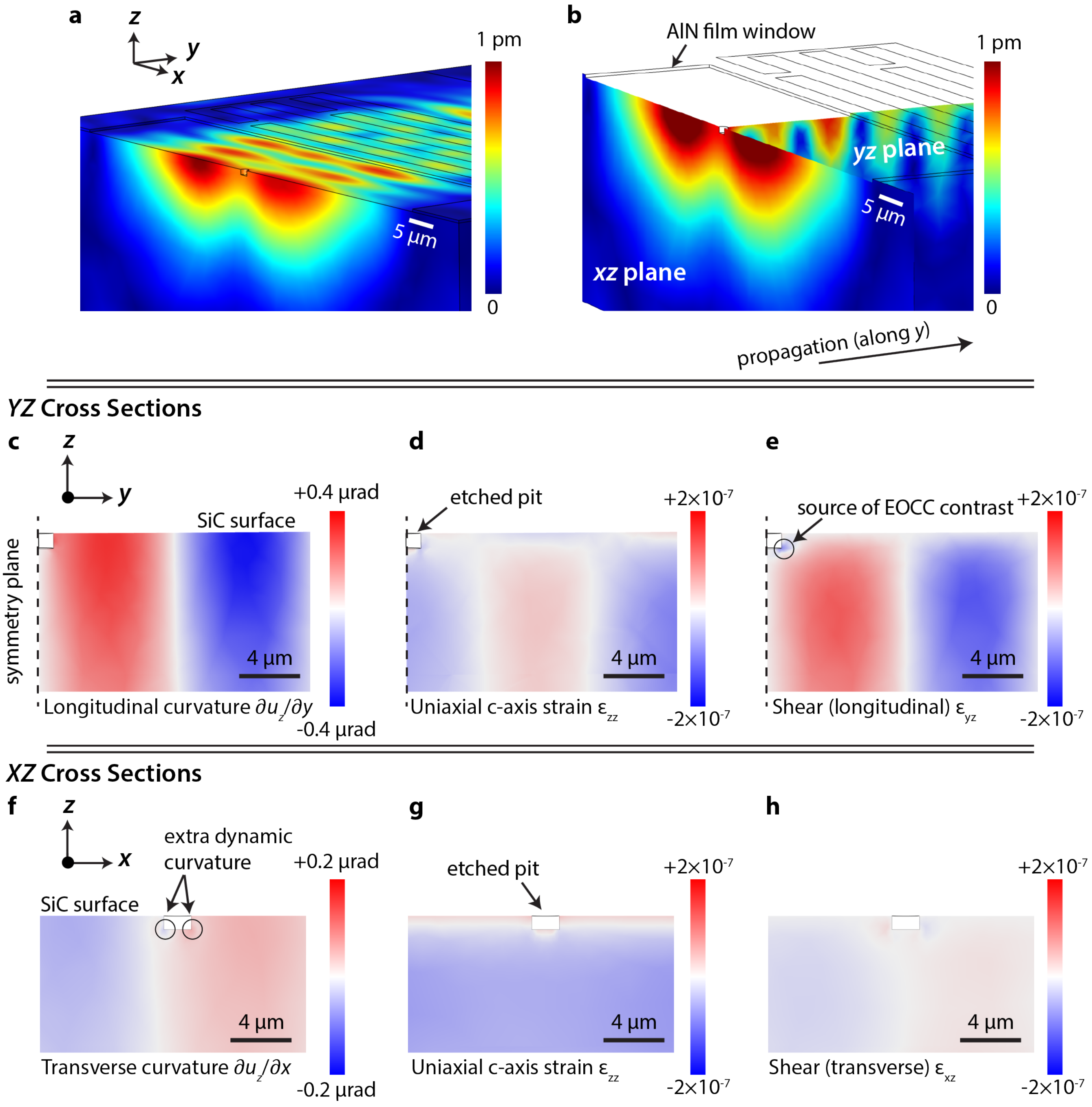

Supplementary Figure 7. **Finite Element Model (COMSOL Multiphysics) in 3D of SAW interacting with pit. 3D View**:(**a**) Displacement profile on the surface of the AlN/SiC. The SAW is generated in the time domain with linear (non-Gaussian shaped) electrodes for simplicity for 4 oscillations. The models boundary conditions (x confinement) and finite planar electrode size distort the SAW transverse mode profile into have two lobes. This is not the case for our experimental Gaussian IDT. (**b**) Cross-sectional view of the SAW illustrating the longitudinal yz-plane and transverse xz-plane. **Longitudinal Cross-Sections (*yz*)**: (**c**) Longitudinal curvature. (**d**) Uniaxial strain along SiC [0001]. (**e**) Shear in the yz-plane (couples piezoelectrically to in-plane electric fields). **Transverse Cross-Sections**: (**f**) Transverse curvature. (**g**) Uniaxial strain along SiC [0001]. (**h**) Shear in the xz-plane (couples piezoelectrically to in-plane electric fields).

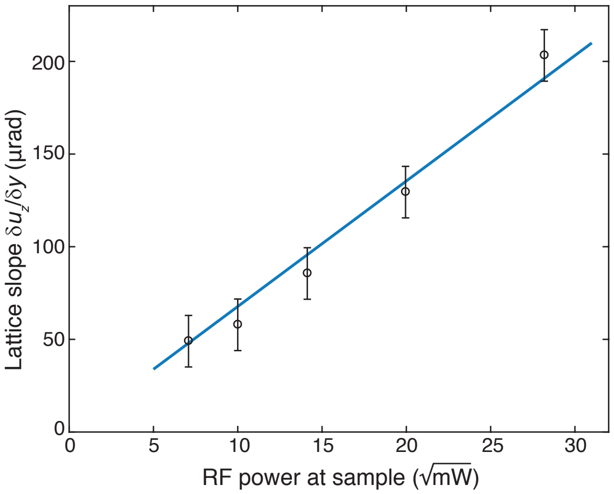

Supplementary Figure 8. **RF power dependence of the SAW detected by s-SXDM.** Linear fit of longitudinal lattice slope ($\delta u_z/\delta y$) to the square-root of applied RF power. This is consistent with SXDM measuring lattice distortions from the SAW displacement and strain amplitude. SXDM measurements are performed at the SAW's maximum curvature near an IDT for consistency. Dynamic lattice slope was obtained from the Bragg peak X centroid standard deviation. Each data point is collected after waiting at least 15 minutes for the sample to thermally stabilize. Error bars are one s.d.

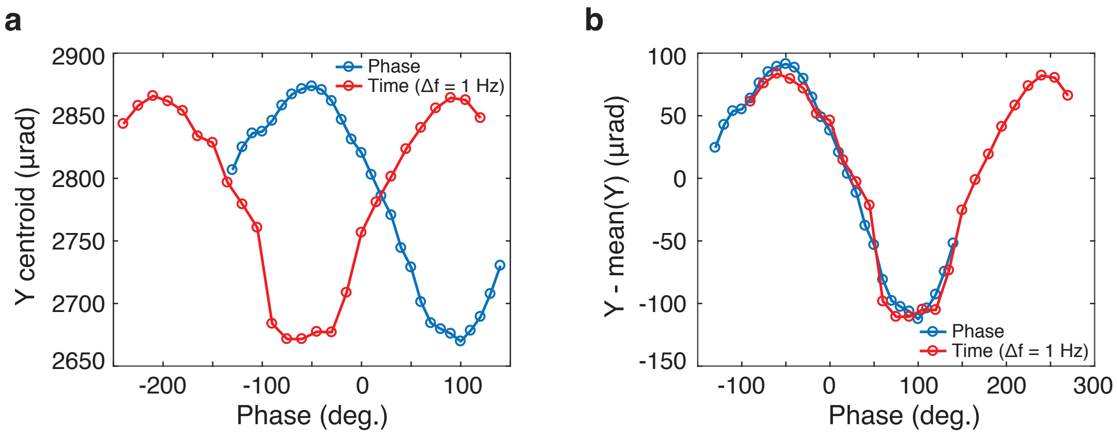

Supplementary Figure 9. **Stroboscopic phase sweeps of the SAW.** (**a**) Analyzed longitudinal X-ray diffraction centroid as a function RF phase. We show results with no frequency detuning while adjusting the signal generator (blue) and results using a 1 Hz frequency detuning with a time delay defining the SAW phase (red). (**b**) Centroid data with the mean subtracted. The red trace has an arbitrary phase offset in order to overlay the two datasets.

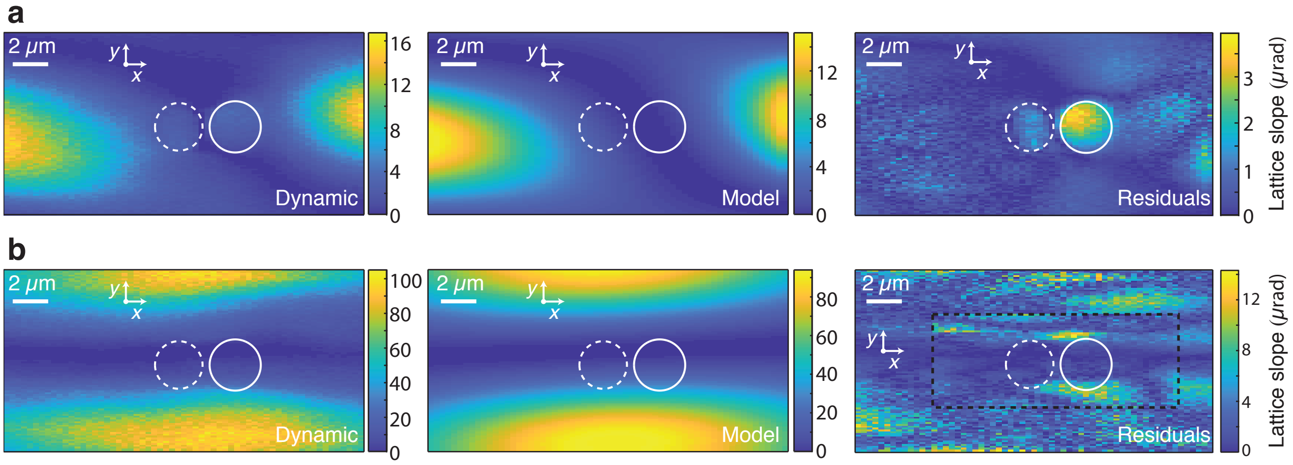

Supplementary Figure 10. **Dynamic transverse and longitudinal curvatures near the structural defect.** From left to right the images are the raw data of stroboscopic SXDM, model of curvature produced by a surface acoustic wave containing a small rotation in-plane, and difference. The pit location is marked by dashed and solid white circles, representing the lower and upper portions the pit (see Fig. 4c). (**a**) Transverse curvature plotted as the lattice slope $\partial u_z/\partial x$. (**b**) Longitudinal curvature plotted as the lattice slope $\partial u_z/\partial y$. A second scan was taken close to the pit for lower noise, which is inset inside the dashed black box.

[1] F. J. Heremans, C. G. Yale, and D. D. Awschalom, Proceedings of the IEEE **104**, 2009 (2016).

[2] G. Wolfowicz, S. J. Whiteley, and D. D. Awschalom, Proceedings of the National Academy of Sciences of the United States of America **115**, 7879 (2018).
